# Dosimetric Verification by Using the ArcCHECK System and 3DVH Software for Various Target Sizes

**DOI:** 10.1371/journal.pone.0119937

**Published:** 2015-03-25

**Authors:** Jin Ho Song, Hun-Joo Shin, Chul Seung Kay, Seok Hyun Son

**Affiliations:** 1 Department of Radiation Oncology, Seoul St. Mary’s Hospital, College of Medicine, The Catholic University of Korea, Seoul, Republic of Korea; 2 Department of Radiation Oncology, Incheon St. Mary’s Hospital, College of Medicine, The Catholic University of Korea, Seoul, Republic of Korea

## Abstract

**Objective:**

To investigate the usefulness of the 3DVH software with an ArcCHECK 3D diode array detector in newly designed plans with various target sizes.

**Methods:**

The isocenter dose was measured with an ion-chamber and was compared with the planned and 3DVH predicted doses. The 2D gamma passing rates were evaluated at the diode level by using the ArcCHECK detector. The 3D gamma passing rates for specific regions of interest (ROIs) were also evaluated by using the 3DVH software. Several dose-volume histograms (DVH)-based predicted metrics for all structures were also obtained by using the 3DVH software.

**Results:**

The isocenter dose deviation was <1% in all plans except in the case of a 1 cm target. Besides the gamma passing rate at the diode level, the 3D gamma passing rate for specific ROIs tended to decrease with increasing target size; this was more noticeable when a more stringent gamma criterion was applied. No correlation was found with the gamma passing rates and the DVH-based metrics especially in the ROI with high-dose gradients.

**Conclusions:**

Delivery quality assurance by using 3DVH and ArcCHECK can provide substantial information through a simple and easy approach, although the accuracy of this system should be judged cautiously.

## Introduction

In order to check the accuracy of radiation delivery in radiotherapy, verification tests such as delivery quality assurance (QA) must be performed prior to patient treatment. Dosimetry verification and QA testing are particularly important in high-precision and complex treatments such as radiosurgery and intensity modulated radiation therapy (IMRT). As these recently developed advanced radiation techniques are characterized according to inherently high dose gradients, even a tiny error can significantly alter the results [1]. Moreover, along with the increasing use of these new radiotherapy techniques, interest in dosimetry verification and QA has been growing.

There are two main methods for evaluating delivery QA: (1) irradiate a plastic phantom that contains an ion-chamber or film with all the beams of the actual treatment plan and measure the actual composite dose; (2) measure each dose of multiple beams by using measuring devices comprising a diode or ion-chamber array. The latter method has been used by several institutions because it can be performed relatively quickly and easily, and can measure all beams. Two-dimensional (2D) detector arrays such as MapCHECK (SunNuclear, Melbourne, FL, USA) and MatriXX (IBA Dosimetry, GmbH, Schwarzenbrook, Germany) were used in the past; these 2D detector array devices had the definite limitation of missing a great portion of the lateral beams because of their individual planar designs [2]. Therefore, three-dimensional (3D) diode array measuring devices such as Delta4 (Scandidos, Uppsala, Sweden) and ArcCHECK (SunNuclear) were recently developed [2–5].

Gamma analysis, which was introduced by Low et al. [6], is used to determine if the measured result is appropriate for comparing and evaluating dose distributions; it uses the following two concepts: dose difference and distance-to-agreement (DTA). The 3% dose difference and 3 mm DTA criterion is commonly used in dosimetric studies as recommended in the American Association of Physicists in Medicine (AAPM) Task Group 119 [7, 8]. When a stricter criterion is required, a 2%/2 mm criterion is often used [4, 9]. The rate that satisfies a gamma criterion is called the gamma passing rate. However, the clinical significance of these criteria is not always clear. For example, even if the gamma passing rate of a specific plan is assumed to be 95%, it cannot ensure that this is actually safer for the patient than another plan with a rate of 85%. Thus, in actual patient treatment, the magnitudes and locations of these dose errors are extremely important.

Several authors recently mentioned that the gamma index obtained through per-beam planar QA can be misleading and insensitive to dosimetric errors [10–12]. In addition, the commercially available 3DVH software from SunNuclear is reported to be able to compensate for the disadvantages. Beam measurements from a treatment plan are input into the 3DVH software, which reconstructs the full 3D dose distribution and allows comparison with the treatment planning system (TPS) calculations. Thus, the 3D distribution of the actually delivered dose can be demonstrated and a dose-volume histogram (DVH) for each target and organ at risk (OAR) can be drawn on the basis of these data [9–13].

Accordingly, in this study, we compared the TPS planned dose to the measured doses by using the ArcCHECK diode array detector and to the predicted doses by using the 3DVH software (2D and 3D gamma analysis) for various target sizes. We also analyzed the 3D DVH-based metrics with the aid of 3DVH software (DVH analysis). Therefore, the usefulness and accuracy of these QA methods has been evaluated.

## Materials and Methods

### Treatment plan design and radiation delivery

**Fig 1:**
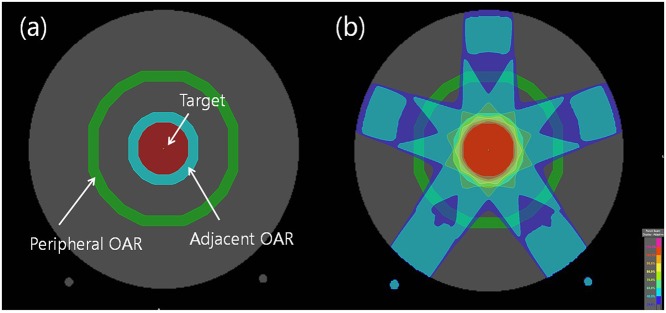
Axial slices of the ArcCHECK CT phantom with the target, adjacent organ at risk (OAR), and peripheral OAR (a). Five portals were used for the planning to cover 95% of the target volume with the prescribed dose. The beam angles were 0°, 72°, 144°, 216° and 288° (b).

Five treatment plans with various target sizes were newly designed by using the computed tomography (CT) images of a homogenous phantom with the same dimensions (26.5 × 26.5 × 27.0 cm^3^) as those of the ArcCHECK (provided by SunNuclear). A circle representing the treatment ‘target’ was contoured with an isocenter as the center of the axial CT image. Targets of different sizes (1, 3, 5, 7, and 9 cm in diameter) for each plan were established (Fig. 1). We also contoured a donut-shaped region of interest (ROI) with 1 cm thickness around the target and named it the ‘adjacent OAR’. The rim of the phantom CT was also contoured in the shape of a donut with a thickness of 1 cm to represent the ‘peripheral OAR’ (Fig. 1). The lengths of the targets and all OARs were set at 5 cm. The volumes per ROI are shown in Table 1.

**Table 1:** Volumes of the target, adjacent organ at risk (OAR) and peripheral OAR with respect to target size.

Target size	Target volume (cc)	Adjacent OAR (cc)	Peripheral OAR (cc)
1 cm	4.2	34.3	238.5
3 cm	38.5	68.3	238.5
5 cm	106.8	102.3	238.5
7 cm	209.2	136.4	238.5
9 cm	345.6	170.4	238.5

OAR: organ at risk

All plans used the iPlan (BrainLAB, v.4.1.2) TPS, and the planning goal was set as 95% coverage of the target by using 3D conformal therapy. Five treatment fields were used with 3 mm multi-leaf collimator (MLC) margins from the target. The beam angles were 0°, 72°, 144°, 216°, and 288°. The chosen prescription doses per each target size plan were 200, 400, 600, 800, and 1000 cGy. A pencil-beam algorithm was used for the dose calculations, with dose grids of 1 mm for both axial and coronal planes.

All tests were carried out on a classic Novalis accelerator (BrainLAB, Feldkirchen, Germany). The Novalis is a linear accelerator system with a single 6 MV energy beam specifically manufactured for high-precision radiosurgery. The MLC of Novalis consists of 26 leaf pairs. The 14 central most leaves have a width of 3 mm, the 6 intermediate leaves have a width of 4.5 mm, and the 6 peripheral leaves are of 5.5 mm width. The maximal field size is 10 × 10 cm^2^. All plans were delivered with their actual treatment angles to the ArcCHECK 3D diode detector. Each plan was measured 3 consecutive times with 5 different radiation doses. Before the radiation delivery, the absolute dose calibration was performed for a 10 × 10 cm^2^ open field at a 100 cm source to skin distance (SSD) according to the TG-51 calibration protocol [14].

### Comparison of isocenter doses

The first validation test involved comparisons of the isocenter doses. The ArcCHECK has a central cavity into which various inserts and detectors can be plugged in and used. An EXRADIN A16-micro ion-chamber (Standard Imaging, Middleton, WI, USA) was inserted in a CavityPlug acrylic insert, which can directly measure isocenter doses. The doses were read by a SUPERMAX electrometer (Standard Imaging). By this method, a directly measured dose from the ion-chamber was compared with the planned isocenter dose. The planned dose was also compared with the predicted dose calculated by the 3DVH software on the basis of the data measured by the ArcCHECK diodes. For comparisons among individual doses, absolute doses were compared among each other; the significance of differences between mean values was determined by independent t-tests. In addition, dose deviations between the measured or predicted dose and planned dose were identified and compared. Dose deviation is expressed as the absolute value, and it was calculated using the following formula:

$$\text{[Dose deviation (\%)=}\frac{\left|\text{Measured or Predicted dose - Planned dose}\right|}{\text{Planned dose}}\times 100(\%)]$$

The difference per target size was evaluated by using one-way analysis of variance (ANOVA). All statistical analyses with p values less than 0.05 were considered significant. Statistical analyses were performed by using STATA/IC ver.12 software (StataCorp, College Station, TX, USA).

### Analysis of 2D and 3D gamma passing rates

#### Analysis of 2D gamma passing rates at the diode level

The ArcCHECK device is a cylindrical water-equivalent phantom with 1,386 diode detectors arranged in a 3D helical fashion at 10 mm intervals within a cylinder with a diameter and length of 21 cm. The angles between the diodes were 5.45 degrees. Each diode in the active detector is 0.8 × 0.8 mm^2^ and is positioned 2.9 cm below the surface. A dedicated software program SNC Patient (v.6.1.1, SunNuclear) was used to analyze the ArcCHECK data. A digital imaging and communications in medicine (DICOM) file for the treatment plan is imported in this software, and a dose grid corresponding to diode detector locations is extracted to compare the calculated and measured doses.

With this software, we obtained the gamma passing rate of each plan at the diode level. The global gamma indices were set as the percentage dose differences calculated with respect to the maximum 3D dose. Two gamma criteria were analyzed, 3%/3 mm and 2%/2 mm, in absolute doses with a threshold of 5%. Whether the gamma passing rate differed as per target size was verified by the one-way ANOVA analysis.

#### Analysis of 3D gamma passing rates for specific regions of interest

One of the advantages of the 3DVH software is that it can calculate the gamma passing rate for the entire radiated volume as well as the gamma passing rate for each corresponding ROI. The data measured in each field by the ArcCHECK diodes are imported into the 3DVH software along with the following 4 other DICOM files from the TPS: the treatment plan, CT images, structures, and calculated dose. The discrepancies between the planned dose and the measured planar dose are calculated, and these calculated errors are back-projected into the original treatment plan. Through this process, we could obtain a newly perturbed 3D dose distribution, which reflects any errors detected by the per-beam planar QA. The proprietary planned dose perturbation (PDP) algorithm was used in the process in this manner [11, 12]. In addition, as the detector pitch between the ArcCHECK diodes was not as dense as those of the planned dose grids, a proprietary interpolation known as Smarterpolation was applied. Through this process, the 3DVH software can calculate the 3D gamma indices for the entire plan as well as the DVH differences for each structure and the 3D gamma indices between the planned and perturbed matrices [11, 12].

With the aid of the 3DVH software, we measured the 3D gamma passing rates for each ROI. The global gamma indices with the two gamma criteria (3%/3 mm and 2%/2 mm) and a threshold of 5% were also used. The 3D gamma passing rates were analyzed according to whether they differed with respect to target size by using the one-way ANOVA and the Pearson correlation analysis.

### Analysis of dose volume histogram (DVH)-based metrics

Various DVH-based predicted metrics for all ROI structures were obtained by using the 3DVH software. The mean dose and the dose covering 50% of the volume (D50) were obtained, and the dose covering 95% of the volume (D95) was calculated for the ‘target’ ROI. These predicted dose matrices for each plan were compared with the planned dose matrices in the TPS. The dose deviation was expressed as the absolute value, and it was defined and calculated as follows:

$$\text{Dose deviation (\%)=}\frac{\left|\text{Predicted dose-Planned dose}\right|}{\text{Planned dose}}\times 100(\%)$$

The dose deviation per target size was analyzed by one-way ANOVA, and the Pearson correlation analysis was performed to examine the correlation between the dose deviation of the DVH-based metrics and the 3D gamma passing rate for each ROI.

## Results

### Comparison of isocenter doses

**Fig 2:**
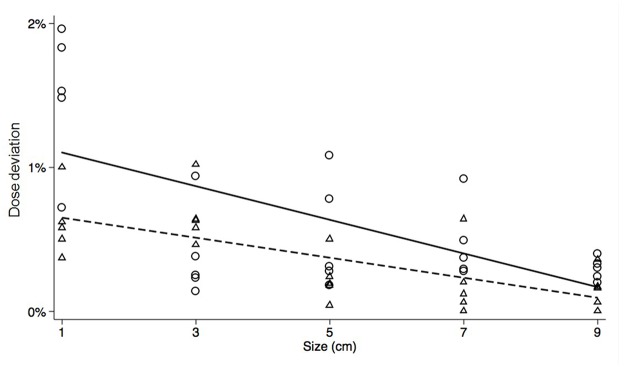
The dose deviation of the isocenter between the 3DVH-predicted dose and the treatment planning system (TPS)-planned dose is plotted as a solid line. The dashed line represents the dose deviation between the ion-chamber measured isocenter dose and planned dose. The dose deviations decreased with increasing target size.

The planned dose at the isocenter was 102.05 ± 1.07% of the prescribed dose. This was not significantly different compared to the dose directly measured by the ion-chamber of 101.81 ± 0.73% (*p* = 0.359) or the predicted dose calculated by the 3DVH software of 102.65 ± 1.41% (*p* = 0.099). Table 2 shows the results of the dose deviations according to target size. With a target size of 1 cm, the differences between measured and planned doses, and predicted and planned doses were 0.61% and 1.50%, respectively, which constituted the largest discrepancy; meanwhile, with a target size of 9 cm, the dose deviations decreased to 0.29% and 0.15%, respectively, constituting the smallest discrepancy (Fig. 2). Every dose deviation was below 1%, except for the case of a target of 1 cm. Thus, the 3DVH software could be used to make relatively precise predictions about isocenter doses, although it is important to be cautious when target size is small.

**Table 2:** Comparison of dose deviations (%) among treatment planning system (TPS)-planned dose, ion-chamber measured isocenter dose, and the 3DVH-predicted isocenter dose.

Target size	1 cm	3 cm	5 cm	7 cm	9 cm	*p value*
Measured dose /Planned dose	0.61 ± 0.24	0.67 ± 0.21	0.23 ± 0.17	0.20 ± 0.25	0.15 ± 0.13	*0.001*
Predicted dose /Planned dose	1.50 ± 0.48	0.39 ± 0.32	0.53 ± 0.39	0.47 ± 0.27	0.29 ± 0.08	*<0.001*

There were significant differences with respect to target size verified by one-way ANOVA.

### Analysis of 2D and 3D gamma passing rates

#### Analysis of 2D gamma passing rates at the diode level

**Fig 3:**
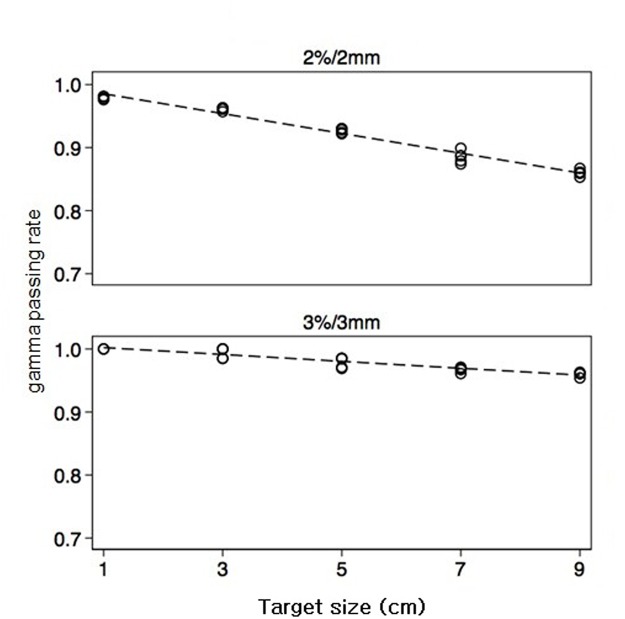
Variations of gamma passing rates at the diode level with various target sizes. The gamma passing rates were calculated with both the 3%/3 mm criterion (lower graph) and the 2%/2 mm criterion (upper graph) in absolute doses with a threshold of 5%. The gamma passing rate decreased as the target size increased.

The gamma passing rates at the diode level are presented in Table 3. For the gamma 3%/3 mm criterion, the gamma passing rates were 100% and 98.5% when the target size was 1 and 3 cm. The gamma passing rates decreased to 96.9%, 96.3% and 95.9% with a target size of 5, 7, and 9 cm respectively, and it was statistically significant (*p <* 0.001). As the target size increased, the gamma passing rates at the diode level tended to decrease; this was more prominent when the more stringent gamma 2%/2 mm criterion was applied (Fig. 3).

**Table 3:** Comparison of gamma passing rates at the diode level according to target size.

Target size	1 cm	3 cm	5 cm	7 cm	9 cm	*p value*
3%/3 mm	100	98.5	96.9 ± 0.1	96.3 ± 0.3	95.9 ± 0.3	*<0.001*
2%/2 mm	97.9 ± 0.2	96.1 ± 0.2	92.6 ± 0.3	88.5 ± 0.9	86.0 ± 0.5	*<0.001*

There were significant differences with respect to target size verified by one-way ANOVA.

#### Analysis of 3D gamma passing rate for specific regions of interest

**Fig 4:**
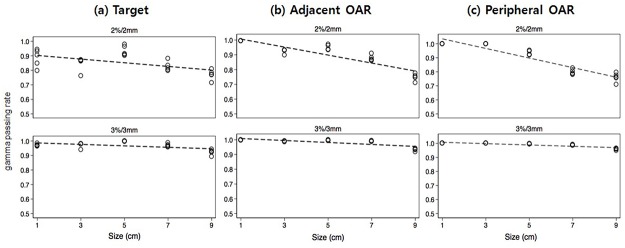
Variation of 3D gamma passing rates for (a) target, (b) adjacent organ at risk (OAR), and (c) peripheral OAR with various target sizes. The gamma passing rates were calculated with both the 3%/3 mm criterion (upper graph) and the 2%/2 mm criterion (lower graph) in absolute doses with a threshold of 5%. The 3D gamma passing rate decreased as the target size increased in all specific regions of interest.

The 3D gamma passing rates for specific ROIs were calculated by using the 3DVH software, and they are presented in Table 4. For the gamma 3%/3 mm criterion, the average 3D gamma passing rates of the target, adjacent OAR, and periphery OAR were 96.6%, 98.1%, and 98.8%, respectively; by using the more stringent gamma 2%/2 mm criterion, the average passing rates of each ROI decreased to 85.2%, 89.8%, and 89.7% respectively. As the target size increased, the gamma passing rates tended to decrease in all ROIs; this was more noticeable when the 2%/2 mm criterion was applied (Fig. 4). The correlation analysis with regard to target size and gamma passing rate of each ROI produced correlation coefficient r values of -0.256 (*p =* 0.010) for the target, -0.667 (*p* < 0.001) for the adjacent OAR, -0.853 (*p* < 0.001) for peripheral OAR, respectively, all of which were consistent with negative correlation.

**Table 4:** Comparison of 3D gamma passing rates of various regions of interest according to target size.

Target size		1 cm	3 cm	5 cm	7 cm	9 cm	*p value*
3%/3 mm	Target	97.1 ± 0.7	97.1 ± 1.7	99.8 ± 0.1	96.8 ± 1.1	92.1 ± 1.8	*<0.001*
	Adjacent	99.8 ± 0.1	99.0 ± 0.2	99.6 ± 0.2	98.9 ± 0.2	93.2 ± 0.8	*<0.001*
	Peripheral	100 ± 0.0	100 ± 0.0	99.6 ± 0.2	98.8 ± 0.6	95.8 ± 1.6	*<0.001*
2%/2 mm	Target	88.5 ± 6.0	84.6 ± 4.6	93.3 ± 3.6	82.6 ± 3.3	76.9 ± 3.4	*<0.001*
	Adjacent	99.4 ± 0.1	92.4 ± 1.4	94.8 ± 1.7	87.6 ± 2.0	74.7 ± 2.3	*<0.001*
	Peripheral	100 ± 0.0	100 ± 0.0	93.2 ± 1.5	79.7 ± 1.9	75.9 ± 3.1	*<0.001*

The differences among the groups were verified by one-way ANOVA.

### Analysis of dose volume histogram (DVH)-based metrics

**Fig 5:**
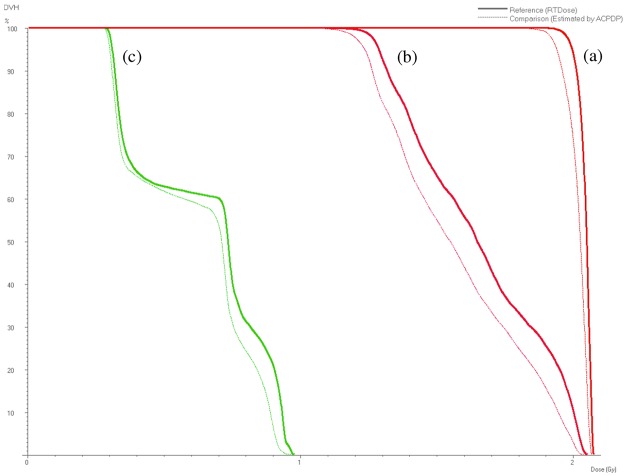
An example of a dose-volume histogram representing the dose distribution for the (a) target, (b) adjacent organ at risk (OAR), and peripheral OAR. The treatment planning system (TPS)-planned doses are plotted as solid lines. The dashed lines represent the 3DVH software- predicted dose, which was calculated on the basis of the ArcCHECK data.

Table 5 shows the % values of planned and predicted doses for each ROI versus the prescribed doses. For the target, the mean dose, D95, and D50 all differed between the planned and predicted doses. On the other hand, the adjacent and peripheral OARs did not differ significantly between the planned and predicted doses. However, in an example of a real DVH curve, some differences can be observed for all ROIs (Fig. 5).

**Table 5:** Comparison of the various dose-volume parameters between the treatment planning system (TPS)-planned dose and the 3DVH-predicted dose.

		Mean dose (%)	*p value*	D95 (%)	*p value*	D50 (%)	*p value*
Target	Plan	101.82 ± 0.32	*<0.001*	99.32 ± 0.47	*<0.001*	102.17 ± 0.32	*0.025*
	3DVH	101.08 ± 0.83		97.15 ± 0.63		101.72 ± 0.90	
Adjacent	Plan	79.54 ± 9.02	*0.108*	N/A		77.58 ± 11.28	*0.209*
	3DVH	75.46 ± 8.60				73.59 ± 10.86	
Peripheral	Plan	32.29 ± 16.45	*0.776*	N/A		30.95 ± 19.21	*0.842*
	3DVH	31.00 ± 15.36				29.89 ± 18.27	

D95: dose covering 95% of the volume; D50: dose covering 50% of the volume; N/A: not assessed. The mean dose is represented as the relative dose compared to the prescribed dose. The statistical significance of the difference between the planned and predicted dos was calculated by independent t-test.

The dose deviations between the planned and the predicted doses of several DVH-based metrics are shown in Table 6. Dose deviations varied in every ROI and every DVH-based dose parameter with respect to varying the target sizes.

**Table 6:** Dose deviation (%) with various dose parameters between the 3DVH-predicted and planned doses with respect to target size.

Target size		1 cm	3 cm	5 cm	7 cm	9 cm	*p value*
Target	mean	0.84 ± 0.49	1.10 ± 0.17	0.78 ± 0.34	1.17 ± 0.18	1.42 ± 0.22	*0.026*
	D95	1.05 ± 0.50	2.72 ± 0.18	2.08 ± 0.33	2.35 ± 0.17	2.71 ± 0.22	*<0.001*
	D50	1.21 ± 0.50	0.70 ± 0.15	0.55 ± 0.35	0.95 ± 0.20	1.20 ± 0.22	*0.011*
Adjacent	mean	5.22 ± 0.26	5.50 ± 0.21	4.49 ± 0.34	5.22 ± 0.21	5.24 ± 0.21	*<0.001*
	D50	5.21 ± 1.13	5.63 ± 0.22	5.70 ± 0.36	4.63 ± 0.21	4.63 ± 0.19	*0.011*
Peripheral	mean	0	0	4.98 ± 0.35	5.08 ± 0.15	4.79 ± 0.22	*<0.001*
	D50	0	0	3.02 ± 0.33	3.99 ± 0.13	4.45 ± 0.22	*<0.001*

D95: dose covering 95% of the volume; D50: dose covering 50% of the volume.

The p values represent the differences among the groups calculated by one-way ANOVA.

Correlation analysis was performed to determine if these dose deviations were related to the 3D gamma passing rates (Table 7). Moderate correlations (i.e., |r| > 0.4) were observed for both the target and peripheral OAR. Here, a negative correlation coefficient indicates the dose deviation decreased with an increasing 3D gamma passing rate of a specific ROI. Therefore, the correlations between the 3D gamma passing rate and DVH-based metrics were comparatively confirmed for the target and peripheral OAR. The correlation increased further with the more stringent gamma criterion (2%/2 mm). However, in terms of the adjacent OAR, the mean dose and D50 showed no correlation, indicating a discrepancy between the 3D gamma passing rate and DVH-based metrics.

**Table 7:** Correlations between the magnitude of dose difference and the gamma passing rate of each region of interest (ROI), calculated with both gamma 3%/3 mm and 2%/2 mm criteria.

		Gamma 3%/3 mm		Gamma 2%/2 mm	
		r	*p value*	r	*p value*
Target	mean	-0.723	*<0.001*	-0.878	*<0.001*
	D95	-0.358	*0.079*	-0.455	*0.022*
	D50	-0.651	*<0.001*	-0.677	*<0.001*
Adjacent	mean	-0.237	*0.253*	-0.268	*0.195*
	D50	0.385	*0.057*	0.418	*0.038*
Peripheral	mean	-0.435	*0.030*	-0.812	*<0.001*
	D50	-0.621	*<0.001*	-0.931	*<0.001*

D95: dose covering 95% of the volume; D50: dose covering 50% of the volume.

Pearson correlation coefficient (r) and two-tailed p-values were calculated.

## Discussion

Radiation delivery QA has traditionally used a composite irradiation method by using an ion-chamber or film, or a beam-by-beam irradiation method by using diode or ion-chamber arrays. However, neither of these methods can show how the delivery dose errors will affect the actual patient treatments [13]. However, the 3DVH software and a 3D diode array detector such as ArcCHECK can be used to obtain a great amount of information quickly and easily. The use of a 3D rather than a 2D diode array allows the data of each beam's eye view to obtained in every field; both entry and exit dose values can be attained as well [3]. By using the 3DVH software, composite data can be produced with the information obtained from each beam. In addition, the information allows us to determine how the composite data affects the dose distribution of an actual ROI. The present study differs from previous 3DVH software studies using virtual measurement data [11, 12] or 2D diode array detector data [10, 13].

Moreover, this study designed the targets, adjacent OAR, and peripheral OAR directly on the CT phantom, in contrast to several previous studies that have used existing patient plans. In this study, the adjacent OAR was contoured close to the target ROI because an important purpose of performing 3D conformal therapy or IMRT is to reduce the radiation dose to the OARs around the treatment target. The target is a high-dose field, and the adjacent ROI is a field with a high dose-gradient. Clinically, this would allude to the relationship of tumor and normal lung in lung cancer treatment or to a metastatic tumor and the spinal cord in bone metastasis for example. Meanwhile, the peripheral OAR measurements are intended to determine accuracy in the low-dose field, for instance at the chest wall or ribs in patients with lung cancer.

The results of the first trial of isocenter dose measurement showed that neither the measured dose nor the 3DVH-predicted dose differed from the planned dose. The largest dose deviation occurred when the target size was 1 cm. However, even in this case, the average dose deviation between the predicted and planned doses was 1.5%; this was the only instance in which the error exceeded 1%. We attribute this difference to the problems of diode measurement itself rather than algorithmic problems in the 3DVH software. According to the study of Kruse [15], the diode can measure a lower dose for smaller fields than the ion-chamber owing to field size effects due to the differential scatter when the diode is calibrated with a field size of 10 × 10 cm^2^. In the present study, as the ArcCHECK was calibrated with a field dimension of 10 × 10 cm^2^, the largest error is thought to have occurred in the plan with a target size of 1 cm. However, the measured dose deviation was less than 0.7% in every instance except that with a field size 1 cm. Therefore, it appears that measurements of the actual isocenter dose by using an ion-chamber in the ArcCHECK can be substituted by the 3DVH predicted isocenter dose in clinical cases in which the target sizes are usually larger than 1 cm.

It is worth noting that when using the ArcCHECK and 3DVH software in this study, the gamma passing rates decreased with increasing target size. This indicates the accuracy varies with respect to field size instead of target size, as the same phenomenon occurred in peripheral OARs with no variations in volume. There are several possible reasons for this, including problems with the TPS algorithm, the ArcCHECK diode or array itself, or the accuracy of the 3DVH algorithm.

The TPS algorithm used in the present study was the pencil-beam algorithm of iPlan RT. The accuracy of the pencil-beam algorithm used in our study has been verified in several studies [16, 17]. Although some studies show the inferior accuracy of the pencil-beam algorithm compared to Monte Carlo algorithms, especially in inhomogeneous conditions [2, 18], no study has demonstrated the inaccuracy of the pencil-beam algorithm according to field size. Therefore, the differences related to field size cannot be explained by the inaccuracy of the TPS algorithm.

Several studies emphasize the accuracy of Smarterpolation and the PDP algorithm in the 3DVH software [9, 11, 13]. In addition to the 3D gamma passing rate for specific ROIs (calculated by the 3DVH software), the gamma passing rate at the diode level (not calculated by the 3DVH software) tended to decrease with increasing target size. Therefore, the differences associated with field size cannot be attributed to the inaccuracy of the 3DVH algorithm.

The results of the present study collectively suggest that the decreasing gamma passing rates with increasing field size are due to the field size dependence of the ArcCHECK diodes. Feygelman et al. showed that differences between the doses measured by the ArcCHECK diode and ion-chamber existed because of varying field sizes [3]. When the ArcCHECK itself is a homogenous phantom, as was the case in the present study, the percentage difference in the measured dose (ArcCHECK diode versus ion-chamber) ranged from -1.1% for a 5 × 5 cm^2^ field to +1.3% for a 25 × 25 cm^2^ field. Li et al. reported similar experimental results: the ArcCHECK diode-measured dose with field sizes increasing from 5 × 5 to 20 × 20 cm^2^ showed errors of 1.2% and 1.7% compared to the TPS-planned dose and ion-chamber measured doses, respectively [4]. Kozelka et al. calibrated the ArcCHECK with a virtual inclinometer algorithm [19]. However, the field size dependence was still observed in our study. It is unclear why the ArcCHECK diode responses are influenced by field sizes. However, Saini and Zhu have suggested that variations in the thickness and design of the buildup around the silicone dye could cause varying field size dependences even for the same diode [20].

It also should be noted that analyses by using gamma indices are not always clinically relevant. The results of several recent studies have indicated that the gamma index is lacking with regard to clinical impact and correlation [10–12, 21]. The present study also failed to demonstrate any definitive correlation between gamma passing rates and DVH-based matrices. In the instances with comparatively homogenous dose distributions, such as the target and peripheral OAR, gamma passing rates were correlated with DVH-based dose parameters; these correlations were stronger with the 2%/2 mm criterion than the 3%/3 mm criterion. On the other hand, regarding the adjacent OAR, no correlations were found between 3D gamma passing rates and DVH-based metrics; instead, the dose deviation decreased with increasing gamma passing rate. This may be because the adjacent OAR represents a field with a high-dose gradient. This situation frequently occurs in clinical cases with inherently high dose gradients, such as IMRT or cases in which normal organs are located close to the target. Therefore, when assessing delivery QA by using ArcCHECK and the 3DVH software, the actual dose distribution must be examined carefully especially in regions with high-dose gradients.

## Conclusions

Delivery QA by using ArcCHECK and the 3DVH software can provide physicians a great deal of information simply and easily, including the previous simple 2D gamma index, the 3D gamma index, the gamma index for each ROI, the perturbed dose distribution, and several DVH-based matrices. The field size dependence of the ArcCHECK diodes requires careful attention; it is recommended that along with the gamma index, various clinically relevant DVH-based matrices should also be examined, especially in regions with high-dose gradients.

## References

[1] Ezzell GA, Galvin JM, Low D, Palta JR, Rosen I, Sharpe MB, et al Guidance document on delivery, treatment planning, and clinical implementation of IMRT: report of the IMRT Subcommittee of the AAPM Radiation Therapy Committee. Med Phys. 2003;30:2089–2115. 12945975 10.1118/1.1591194

[2] Petoukhova AL, van Egmond J, Eenink MG, Wiggenraad RG, van Santvoort JP. The ArcCHECK diode array for dosimetric verification of HybridArc. Phys Med Biol. 2011;56:5411–5428. 10.1088/0031-9155/56/16/021 21804180

[3] Feygelman V, Zhang G, Stevens C, Nelms BE. Evaluation of a new VMAT QA device, or the "X" and "O" array geometries. J Appl Clin Med Phys. 2011;12:3346 21587178 10.1120/jacmp.v12i2.3346 PMC5718675

[4] Li G, Zhang Y, Jiang X, Bai S, Peng G, Wu K, et al Evaluation of the ArcCHECK QA system for IMRT and VMAT verification. Phys Med. 2012;29:295–303. 10.1016/j.ejmp.2012.04.005 22583979

[5] Bedford JL, Lee YK, Wai P, South CP, Warrington AP. Evaluation of the Delta4 phantom for IMRT and VMAT verification. Phys Med Biol. 2009;54:N167–176. 10.1088/0031-9155/54/9/N04 19384007

[6] Low DA, Harms WB, Mutic S, Purdy JA. A technique for the quantitative evaluation of dose distributions. Med Phys. 1998;25:656–661. 9608475 10.1118/1.598248

[7] Ezzell GA, Burmeister JW, Dogan N, LoSasso TJ, Mechalakos JG, Mihailidis D, et al IMRT commissioning: multiple institution planning and dosimetry comparisons, a report from AAPM Task Group 119. Med Phys. 2009;36:5359–5373. 19994544 10.1118/1.3238104

[8] Nelms BE, Simon JA. A survey on planar IMRT QA analysis. J Appl Clin Med Phys. 2007;8:2448 17712302 10.1120/jacmp.v8i3.2448 PMC5722600

[9] Nelms BE, Opp D, Robinson J, Wolf TK, Zhang G, Moros E, et al VMAT QA: measurement-guided 4D dose reconstruction on a patient. Med Phys. 2012;39:4228–4238. 10.1118/1.4729709 22830756

[10] Carrasco P, Jornet N, Latorre A, Eudaldo T, Ruiz A, Ribas M. 3D DVH-based metric analysis versus per-beam planar analysis in IMRT pretreatment verification. Med Phys. 2012;39:5040–5049. 10.1118/1.4736949 22894429

[11] Zhen H, Nelms BE, Tome WA. Moving from gamma passing rates to patient DVH-based QA metrics in pretreatment dose QA. Med Phys. 2011;38:5477–5489. 10.1118/1.3633904 21992366

[12] Nelms BE, Zhen H, Tome WA. Per-beam, planar IMRT QA passing rates do not predict clinically relevant patient dose errors. Med Phys. 2011;38:1037–1044. 21452741 10.1118/1.3544657 PMC3188652

[13] Olch AJ. Evaluation of the accuracy of 3DVH software estimates of dose to virtual ion chamber and film in composite IMRT QA. Med Phys. 2012;39:81–86. 10.1118/1.3666771 22225277

[14] Almond PR, Biggs PJ, Coursey BM, Hanson WF, Huq MS, Nath R, et al AAPM's TG-51 protocol for clinical reference dosimetry of high-energy photon and electron beams. Med Phys. 1999;26:1847–1870. 10505874 10.1118/1.598691

[15] Kruse JJ. On the insensitivity of single field planar dosimetry to IMRT inaccuracies. Med Phys. 2010;37:2516–2524. 20632563 10.1118/1.3425781

[16] Ahnesjo A, Saxner M, Trepp A. A pencil beam model for photon dose calculation. Med Phys. 1992;19:263–273. 1584117 10.1118/1.596856

[17] Bortfeld T, Schlegel W, Rhein B. Decomposition of pencil beam kernels for fast dose calculations in three-dimensional treatment planning. Med Phys. 1993;20:311–318. 8497215 10.1118/1.597070

[18] Ali I, Ahmad S. Quantitative assessment of the accuracy of dose calculation using pencil beam and Monte Carlo algorithms and requirements for clinical quality assurance. Med Dosim. 2013;38:255–261. 10.1016/j.meddos.2013.02.005 23558145

[19] Kozelka J, Robinson J, Nelms B, Zhang G, Savitskij D, Feygelman V. Optimizing the accuracy of a helical diode array dosimeter: a comprehensive calibration methodology coupled with a novel virtual inclinometer. Med Phys. 2011;38:5021–5032. 10.1118/1.3622823 21978046

[20] Saini AS, Zhu TC. Energy dependence of commercially available diode detectors for in-vivo dosimetry. Med Phys. 2007;34:1704–1711. 17555252 10.1118/1.2719365

[21] Chan MF, Li J, Schupak K, Burman C. Using a Novel Dose QA Tool to Quantify the Impact of Systematic Errors Otherwise Undetected by Conventional QA Methods: Clinical Head and Neck Case Studies. Technol Cancer Res Treat. 2013;13:57–67. 10.7785/tcrt.2012.500353 23819494

